# Input-Independent Homeostasis of Developing Thalamocortical Activity

**DOI:** 10.1523/ENEURO.0184-21.2021

**Published:** 2021-05-19

**Authors:** Pouria Riyahi, Marnie A. Phillips, Matthew T. Colonnese

**Affiliations:** 1 Department of Biomedical Engineering, The George Washington University, Washington DC 20052; 2 Department of Pharmacology and Physiology, The George Washington University, Washington DC 20052

**Keywords:** activity, EEG, homeostasis, retinal waves, spindle-burst, visual cortex

## Abstract

The isocortex of all mammals studied to date shows a progressive increase in the amount and continuity of background activity during early development. In humans the transition from a discontinuous (mostly silent, intermittently bursting) cortex to one that is continuously active is complete soon after birth and is a critical prognostic indicator. In the visual cortex of rodents this switch from discontinuous to continuous background activity occurs during the 2 d before eye-opening, driven by activity changes in relay thalamus. The factors that regulate the timing of continuity development, which enables mature visual processing, are unknown. Here, we test the role of the retina, the primary input, in the development of continuous spontaneous activity in the visual cortex of mice using depth electrode recordings from enucleated mice *in vivo*. Bilateral enucleation at postnatal day (P)6, one week before the onset of continuous activity, acutely silences cortex, yet firing rates and early oscillations return to normal within 2 d and show a normal developmental trajectory through P12. Enucleated animals showed differences in silent period duration and continuity on P13 that resolved on P16, and an increase in low frequency power that did not. Our results show that the timing of cortical activity development is not determined by the major driving input to the system. Rather, even during a period of rapid increase in firing rates and continuity, neural activity in the visual cortex is under homeostatic control that is largely robust to the loss of the primary input.

## Significance Statement

Uncovering the mechanistic underpinnings of electroencephalography (EEG) development is critical to increasing the diagnostic potential of this cheap and portable methodology. An important component of this maturation is the acquisition of activity that is continuous, i.e., lacking silent periods. Here, we used background activity in the visual cortex of developing unanesthetized mice to show that the primary sensory input plays little role in the development of continuity and normal firing rates, which instead appear to be regulated by mechanisms internal to thalamus and cortex. These findings suggest that damage to driving thalamic inputs will be difficult to detect by EEG, and point to the importance of firing rate homeostasis in regulating even early development.

## Introduction

Spontaneous activity, sometimes called background or resting-state, is a pervasive feature of thalamocortical circuit function (Raichle, 2010; Uddin, 2020). The patterns of this activity are both a target and effector of arousal state modulation (McCormick et al., 2020). Multiple roles have been suggested for spontaneous activity: mediating attention, increasing signal-to-noise, resetting synaptic weights, and changing the functional connectivity of neurons (Harris and Thiele, 2011; Froudarakis et al., 2019; Tononi and Cirelli, 2020). Thus, it is not surprising that the acquisition of normal background activity is a key developmental checkpoint (Pavlidis et al., 2017). While phenomenologically well characterized in humans and animals (Tuge et al., 1960; Gramsbergen, 1976; Vanhatalo and Kaila, 2006; André et al., 2010), the circuit basis of background activity development is poorly understood (Wallois et al., 2020). This knowledge is crucial for treatment of early brain disorders and to improve the diagnostic utility of electroencephalography (EEG) for neonatal medicine (Pavlidis et al., 2017).

The background EEG of human infants fully resembles adult sleep-wake patterns a few months after birth, when sleep-spindles and large-amplitude slow-waves emerge (Tiriac and Blumberg, 2016; Dereymaeker et al., 2017). Premature infants produce cortical activity that is highly discontinuous, with bursts of activity interrupted by long silent intervals of up to 60 s, which become shorter with age. These periods of activity consist of unique early patterns such as δ-brushes, tracé alternate, and temporal theta, the occurrence of which is both age and region dependent (Whitehead et al., 2017; Wallois et al., 2020). Neonatal injury, such as hypoxia/ischemia, can reverse gains in continuity (Stevenson et al., 2017), the recovery of which is a positive prognostic for neonatal brain function (van Rooij et al., 2005; Iyer et al., 2014).

All mammalian species yet examined show a similar developmental trajectory (Cirelli and Tononi, 2015). The rodent visual system has proven to be a good model of cortical activity development, as the timing of critical events has been aligned to humans (Colonnese and Phillips, 2018) and control of the inputs can be easily achieved (Leighton and Lohmann, 2016; Seabrook et al., 2017). In the primary visual cortex (V1) of rats and mice, spontaneous activity is discontinuous throughout most of the first two postnatal weeks, rapidly becoming continuous between P11 and P13, just before eye-opening (Colonnese et al., 2010; Shen and Colonnese, 2016). This switch in the macropatterning of background activity is contemporaneous with a change in the spectral patterns of activity as well as an increase in neural firing rates, reflecting an end to immature oscillations and the emergence of the adult-like “active” state, as well as robust sleep-wake rhythms (Colonnese, 2014).

Similar changes appear to occur in all thalamocortical pathways studied to date (Colonnese and Phillips, 2018; Hanganu-Opatz et al., 2021), but the mechanisms are unknown. Recent recordings in the visual relay thalamus, the dorsal lateral geniculate nucleus (dLGN), suggest that changes there cause the maturation of the background EEG in V1 (Murata and Colonnese, 2018). However, activity of the major input to dLGN, retinal ganglion cells (RGCs), also becomes more continuous around eye-opening (Demas et al., 2003). Thus, it is possible that the acquisition of adult-like background activity in dLGN and V1 simply reflects retinal maturation.

Here, we examine the role of retina in timing the development of background activity in V1 by removing both eyes during the early period of discontinuity and recording V1 activity development through the eye-opening period. Despite subtle differences in enucleated animals, the gross acquisition of continuous background activity is very similar in enucleated and control. We conclude that powerful homeostatic mechanisms within thalamocortex control activity development in V1, and that a genetically programmed shift within thalamus (or its modulatory inputs) controls the timing of cortical background activity development, without instruction from the retina.

## Materials and Methods

### Animal care

Animal care and procedures were in accordance with *The Guide for the Care and Use of Laboratory Animals* (National Institutes of Health) and approved by the Institutional Animal Care and Use Committee at The George Washington University. Postnatal day (P)0 is the day of birth. C57BL/6 were obtained from Hilltop Lab Animals as timed pregnant females, and kept in a designated, temperature and humidity-controlled room on a 12/12 h light/dark cycle and examined once per day for pups. For bilateral enucleations, carprofen (20 mg/kg) in saline was injected 1 h before surgery to reduce pain and inflammation. Surgical anesthesia was induced with 3% isoflurane vaporized in 100% O_2_, verified by tail-pinch. An incision was made in the eyelid (P6) and the globe of the eye was removed using forceps. The eye socket was filled with sterile surgical foam (GelFoam) and the eyelid closed using a tissue adhesive (Vetbond). Pups were postoperatively monitored and received follow-up injections of carprofen daily for 2 d. Sham control animals received identical treatment, including eyelid puncture with the tip of a suture needle, without enucleation or GelFoam.

### *In vivo* electrophysiology

Carprofen (20 mg/kg) in saline was injected 1 h before surgery to reduce pain and inflammation. Surgical anesthesia was induced with 3% isoflurane vaporized in 100% O_2_, verified by toe-pinch, then reduced to 1.5–3% as needed by monitoring breathing rate and toe pinch response. An electrical heating pad (36°C) provided thermoreplacement. For attachment of the head-fixation apparatus, the scalp was excised to expose the skull, neck muscles were detached from the occipital bone, and the membranes were removed from the surface of the skull. Topical analgesic was applied to the incision of animals older than P8 (2.5% lidocaine/prilocaine mix, Hi-Tech Pharmacy Co). Application to younger animals was lethal. The head-fixation apparatus was attached to the skull with grip cement (Dentsply) over Vetbond tissue adhesive (3 M). The fixation bar consisted of a custom manufactured rectangular aluminum plate with a central hole for access to the skull. After placement, the animal was maintained with 0.5–1% isoflurane until the dental cement cured, after which it recovered on a warming table.

For recording, animals were head-fixed via the plate. Body movements were restricted by placement in a padded tube. Body temperature was monitored via thermometer placed under the abdomen, and maintained above 33°C via thermocoupled heating pad (FHC). Body motion was monitored with a piezoelectric device placed below the restraint tube. For electrode access, a craniotomy was performed, thinning the skull if necessary, and resecting small bone flaps, to produce a small opening (∼150–300 μm in diameter). V1 was targeted by regression of adult brain λ-bregma distances: 1.5–2.5 mm lateral and 0.0–0.5 mm rostral to λ. All recordings were made using a single shank, 32 channel array arranged in two parallel lines of contacts (A1x32-Poly2-5 mm-50s-177, NeuroNexus Technologies). The electrode penetrated the brain orthogonally to the surface and was advanced to a depth of 500–800 μm using stereotaxic micromanipulator until the top channels touched the cerebral-spinal fluid. Isoflurane was withdrawn and the animal acclimated to the setup for at least 60 min before recording. Unless otherwise noted recordings lasted 30 min. All recording was performed in the dark (<0.01 lumens).

For acute enucleation, after baseline recording, surgical anesthesia was reintroduced on the rig with the electrode in place and the eyes removed as described above.

Following recordings, all animals were killed by anesthetic overdose followed by decapitation.

### Data acquisition and analysis

Data were amplified 192V/V and digitized at 30 kHz on SmartLink Headstage and recorded with the SmartBox 1.0 (Neuronexus). Recordings were imported in MATLAB using Neuronexus supplied macros and custom code. Depth (d)EEG signals were derived by down-sampling wide-band signal to 1 kHz after application of 0.1- to 350-Hz zero-phase low-pass filter. To remove common-mode noise and volume conduction from sub-cortical structures, signals were referenced to a contact in or just below layer 6 with minimal spiking. For multiunit activity, channels with high noise (outside the brain or bad contacts) were manually eliminated and the remaining channels saved as binary structures for spike sorting using Kilosort (Pachitariu et al., 2016). To derive “cleaned” multiunit activity, clusters were identified using the default parameters except for ops.Th = [3 6 6]. Noise clusters were manually removed using the spike sorting GUI Phy (Rossant et al., 2016) and all remaining clusters were collapsed by central contact as determined by the mean spike-waveform minima. Spike times were binned at 1 ms.

All analyses were performed in MATLAB. Recordings were not included in the analysis if they contained spreading depression (*n* = 4) or if the merged L2–L4 or L5–L6 multi-unit activity (MUA) was below 0.1 Hz (*n* = 7). Spreading depression was identified as a sawtooth oscillation that spread slowly from surface to deep layers over 10–20 s and was followed by two or more minutes of silence. One additional animal was eliminated because the file was corrupt and could not be imported. Before analysis, periods of movement as identified by the piezo signal, were removed. Channels were divided into superficial (L2–L4) and deep (L5–L6). Superficial layers were identified by depth and the presence of high-frequency (>10 Hz) power (Colonnese and Khazipov, 2010). Spike rates and continuity were derived from the 4 channels in each (L2–L4 and L5–L6) with the highest mean spike rate. For spectral analysis, a contact in the center of L2–L4 was selected. Spectral decomposition of the dEEG signal used the multitaper method (Mitra and Bokil, 2007). Spectra were calculated for 1s windows (time-bandwidth product three and number of tapers 5). Window width and time-bandwidth products were chosen empirically to maximize the spindle-burst frequencies in young animals. Normalized mean power for each animal was calculated by averaging all windows during non-movement periods then dividing by mean 1- to 60-Hz power. Division into active and inactive periods followed loosely the method of Renart et al. (2010) as implemented previously in neonatal mice (Colonnese et al., 2017). The smoothed multiunit spike rates were calculated by applying a Gaussian window with 25-ms half-width to the summed MUA for each layer. Based on this smoothed spike-rate vector, active and inactive periods were identified with a threshold set to 10% of maximal spike rate for that mouse and layer. Inactive periods of <100 ms were folded into the adjacent active periods. For a subset of analyses (Fig. 4*C*) the Gaussian half-width was increased to 200 ms. For analysis of active and silent period distributions, the occurrence of each duration was accumulated into one of 50 bins with log distributed widths, between 10^1^ and 10^5^ ms, and normalized to the total occurrences. To quantify the change in distributions between days as a single variable, we calculated the absolute difference between the normalized distributions for each animal in each group against each of the animals in the reference group (P16–P17). For each group and age these pair-wise differences were averaged.

### Statistical procedures

All data are mean ± SD, except for the distribution differences which are 95% confidence intervals. The statistical test applied is noted along with results. Statistical tests were applied in MATLAB using the inbuilt functions (*ttest2*, *anovan*). Significance statements regarding normalized frequency power distributions were calculated by permutation analysis following the permutation test method of Cohen (Cohen, 2014), using custom macros. Frequency resolution was 1 Hz from 1 to 60 Hz. The significance threshold was *p* < 0.05.

## Results

### P6 enucleation suppresses activity in visual cortex

**Figure 1.**
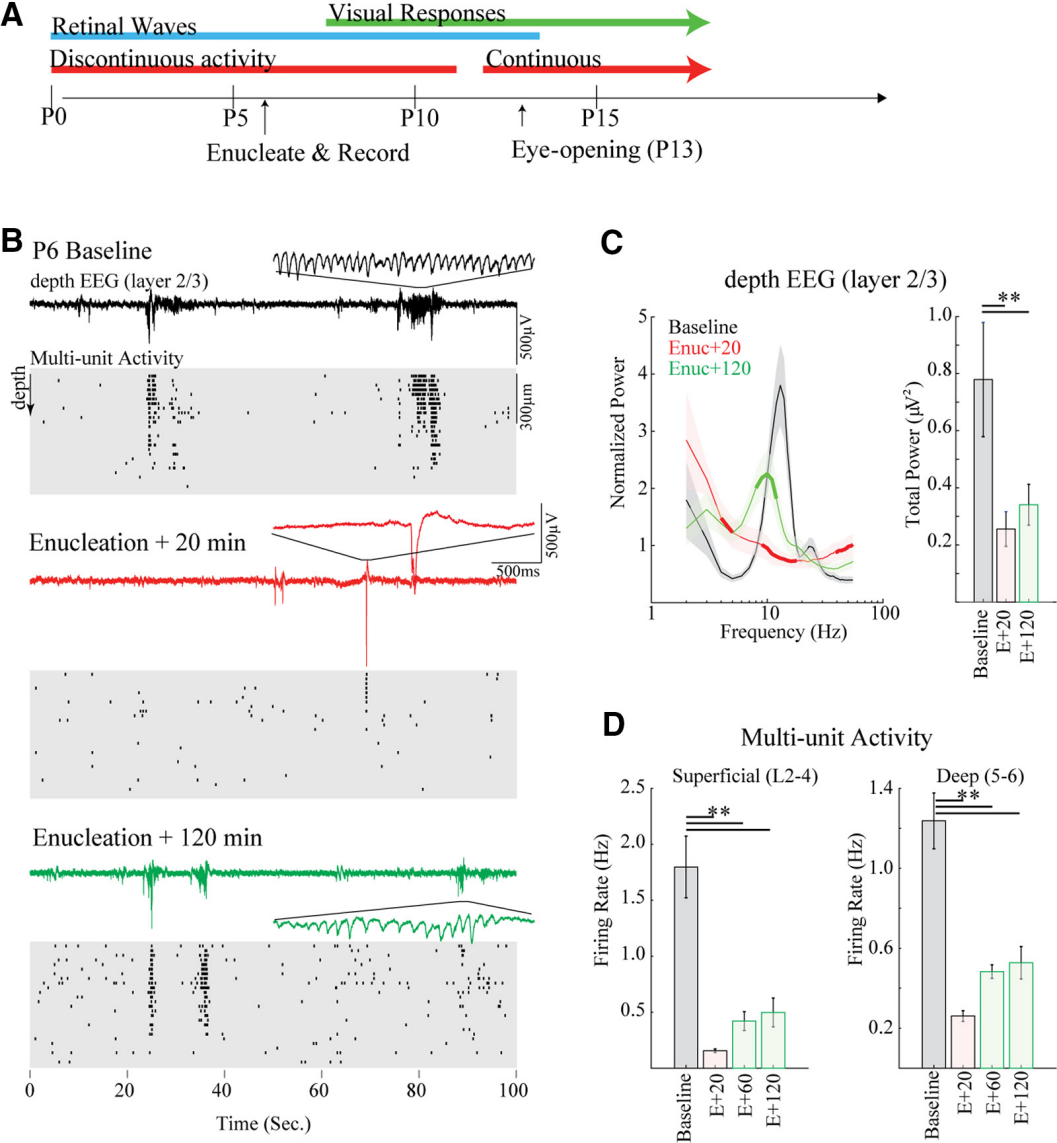
Enucleation acutely reduces activity and spindle-bursts in mouse visual cortex. ***A***, Overview of major developmental stages in visual cortex of the mouse relative to acute enucleation on the sixth postnatal day (P6). ***B***, Representative example of spontaneous activity before (baseline), 20 and 120 min following bilateral enucleation. Top line shows depth EEG, with detail of one spindle-burst. Cleaned multiunit raster of spike times is below. Each line is from a single contact with 25 μm between contacts. Note rapid loss of rapid oscillations in bursts as well as spontaneous spiking at Enuc + 20 min, with some recovery of both by Enuc + 120 min. ***C***, Population mean (*n* = 5) spectral analysis for dEEG. Left, Mean ± SD spectral power distribution. Thick line segment shows regions of significant difference from preceding condition (i.e., baseline for Enuc + 20, and Enuc + 20 for Enuc + 120) determined by permutation analysis. Right, Summed 1- to 60-Hz power. Animals lose and then recover spindle-burst frequencies following enucleation, but total power remains low. ***D***, Population mean of multiunit firing rates; **p < 0.01 by Tukey’s *post hoc* test.

Early enucleations were made at P6. This age was chosen because it is the earliest age when enucleation does not produce gross sprouting of other sensory inputs to dLGN or cause reorganization of corticocortical connections (Olavarria and Hiroi, 2003). We first assayed the acute effects of early bilateral enucleation on spontaneous activity in the visual cortex of mice. Recordings were made in five P6 mice (two litters) using a single-shank 32-channel multielectrode array inserted in the monocular zone of V1. After baseline recordings, the mice were anesthetized and binocular enucleation performed with the electrode in place (Fig. 1*A*).

As previously described (Shen and Colonnese, 2016), baseline spontaneous activity on P6 was largely restricted to regularly repeating 1- to 10-s periods of high-firing rates and prominent 10- to 20-Hz oscillations in the superficial layers (Fig. 1*B*,*C*). These oscillatory periods, called slow-activity transients (Colonnese and Khazipov, 2010), consist of multiple spindle-bursts which are the result of spontaneous retinal wave input to the thalamus (Murata and Colonnese, 2016), which is then conveyed to topographically appropriate locations in visual cortex (Ackman and Crair, 2014; Kummer et al., 2016; Leighton and Lohmann, 2016). After enucleation, spontaneous cortical activity was recorded for 2 h (Fig. 1*B*) and analyzed in 20-min windows beginning 20, 60, and 120 min after enucleation (Table 1). Previous studies examining recovery time in non-enucleated rats indicated that 10–15 min is sufficient for recovery from the effects of anesthesia (Colonnese, 2014). Immediately after recovery from anesthesia (E + 20 min), spindle-bursts were absent as evidenced by an elimination of the prominent 10- to 18-Hz bump in the normalized frequency power of the layer 2/3 depth (d)EEG (local field potential; Fig. 1*C*). Spontaneous activity overall was severely curtailed as well, as evidenced by a threefold drop in 1- to 60-Hz power (Table 1). Likewise, the multiunit firing rate dropped by 90% in superficial layers and 79% in deep layers. The remaining activity consisted of isolated firing and short (<1 s) bursts. Such a dramatic loss of activity is similar to that observed following retinal activity blockade in rats by microinjection at similar ages (Murata and Colonnese, 2016), suggesting that it is because of the loss of RGC activity, not unexpected side-effects of retinal trauma. Total spontaneous activity remained suppressed at 60 and 120 min after enucleation. By 120 min, however, the cortex had begun to produce spindle-burst oscillations (Fig. 1*B*), and as a result, we observed a significant increase in normalized frequency power 8–15 Hz (Fig. 1*C*), although the total 1- to 60-Hz power remained similar to 20 min after enucleation. Mean firing rates tripled between 20 and 120 min after enucleation in superficial layers and doubled in deep layers (Fig. 1*D*), though this increase was not significant by *post hoc* test, likely because of the high total variability introduced by preenucleation firing rates.

**Table 1:** Descriptive and inferential statistics by figure

Figure 1*C*	μV2		Figure 1*D*	Spikes/s			Figure 5*B*		
Baseline	78 ± 9			Superficial	Deep			Superficial	Deep
E + 20	26 ± 3		Baseline	1.80 ± 0.61	1.24 ± 0.31		Sham (spike/s)	5.76 ± 4.71	6.44 ± 4.22
E + 120	34 ± 3		E + 20	0.16 ± 0.04	0.26 ± 0.06		Eunucleated	4.56 ± 3.63	6.9 ± 4.91
			E + 60	0.42 ± 0.19	0.48 ± 0.08		Sham (Cont.)	0.73 ± 0.22	0.87 ± 0.14
			E + 120	0.50 ± 0.29	0.53 ± 0.18		Enucleated	0.64 ± 0.25	0.78 ± 0.17
									
Figure 3*B*	log10(spikes per second)								
Superficial	P8–P9	P10	P11	P12	P13	P14	P15	P16–P17	ANOVA
Sham	−0.43 ± 0.49	−0.03 ± 0.34	0.16 ± 0.40	0.25 ± 0.19	0.29 ± 0.22	0.66 ± 0.26	0.76 ± 0.22	0.87 ± 0.13	Age *F* = 63.5 *p* < 10^−10^
Enucleated	−0.37 ± 0.36	−0.18 ± 0.16	−0.10 ± 0.05	0.40 ± 0.14	0.52 ± 0.19	0.49 ± 0.19	0.73 ± 0.24	1.07 ± 0.10	Group *F* = 1.18 *p* = 0.30
									Layer *F* = 1.57 *p* = 0.21
Deep									A × G *F* = 2.03 *p* = 0.06
Sham	−0.51 ± 0.29	−0.02 ± 0.25	0.24 ± 0.33	0.33 ± 0.26	0.55 ± 0.44	0.87 ± 0.18	0.10 ± 0.07	0.95 ± 0.18	A × L *F* = 0.96 *p* = 0.47
Enucleated	−0.53 ± 0.32	−0.12 ± 0.29	−0.06 ± 0.15	0.40 ± 0.40	0.76 ± 0.32	0.61 ± 0.23	0.61 ± 0.20	0.90 ± 0.09	G × L *F* = 1.63 *p* = 0.20
									A × G × L *F* = 0.26 *p* = 0.97
									
Figure 3*C*	Continuity								
Superficial	P8–P9	P10	P11	P12	P13	P14	P15	P16–P17	
Sham	0.11 ± 0.06	0.19 ± 0.05	0.24 ± 0.13	0.40 ± 0.20	0.47 ± 0.12	0.77 ± 0.19	0.78 ± 0.15	0.74 ± 0.19	Age *F* = 61.47 *p* < 10^−10^
Enucleated	0.09 ± 0.01	0.13 ± 0.04	0.17 ± 0.04	0.45 ± 0.11	0.41 ± 0.05	0.36 ± 0.09	0.41 ± 0.16	0.70 ± 0.11	Group *F* = 12.85 *p* = 0.0005
									Layer *F* = 39.21 *p* = 10^−8^
Deep									A × G *F* = 6.97 *p* = 10^−6^
Sham	0.13 ± 0.07	0.29 ± 0.12	0.32 ± 0.08	0.45 ± 0.11	0.72 ± 0.28	0.94 ± 0.07	0.96 ± 0.04	0.87 ± 0.16	A × L *F* = 3.24 *p* = 0.0035
Enucleated	0.09 ± 0.05	0.25 ± 0.16	0.24 ± 0.10	0.52 ± 0.16	0.92 ± 0.11	0.65 ± 0.25	0.67 ± 0.22	0.87 ± 0.08	G × L *F* = 1.71 *p* = 0.19
									A × G × L *F* = 0.44 *p* = 0.88
									
Figure 4*B*	Distance vs P16–P17 distribution								
Superficial	P8–P9	P10–P11		P12	P13	P14	P15	P16–P17	
Sham	0.57 ± 0.05	0.49 ± 0.02		0.51 ± 0.05	0.43 ± 0.03	0.45 ± 0.04	0.29 ± 0.02	0.27 ± 0.09	Age *F* = 28.19 *p* < 10^−10^
Enucleated	0.62 ± 0.08	0.52 ± 0.05		0.42 ± 0.04	0.38 ± 0.03	0.37 ± 0.02	0.49 ± 0.06	0.38 ± 0.08	Group *F* = 6.38 *p* = 0.0118
									Layer *F* = 14.29 *p* = 0.0002
Deep									A × G *F* = 2.20 *p* = 0.06
Sham	0.79 ± 0.07	0.48 ± 0.03		0.63 ± 0.08	0.55 ± 0.06	0.52 ± 0.05	0.42 ± 0.04	0.20 ± 0.09	A × L *F* = 3.11 *p* = 0.0053
Enucleated	0.82 ± 0.06	0.64 ± 0.05		0.57 ± 0.06	0.55 ± 0.06	0.52 ± 0.03	0.53 ± 0.05	0.39 ± 0.08	G × L *F* = 0.48 *p* = 0.49
									A × G × L *F* = 1.17 *p* = 0.32
									
Figure 5*B*	Distance vs P16–P17 distribution								
Superficial	P8–P9	P10–P11		P12	P13	P14	P15	P16–P17	
Sham	0.68 ± 0.02	0.70 ± 0.03		0.83 ± 0.07	0.49 ± 0.03	0.35 ± 0.05	0.29 ± 0.02	0.20 ± 0.07	Age *F* = 69.16 *p* < 10^−10^
Enucleated	0.72 ± 0.05	0.77 ± 0.07		0.36 ± 0.03	0.41 ± 0.05	0.41 ± 0.03	0.48 ± 0.09	0.32 ± 0.09	Group *F* = 11.06 *p* = 0.0009
									Layer *F* = 2.28 *p* = 0.13
Deep									A × G *F* = 13.28 *p* < 10^−10^
Sham	0.78 ± 0.04	0.64 ± 0.04		0.75 ± 0.07	0.51 ± 0.04	0.36 ± 0.04	0.29 ± 0.03	0.29 ± 0.07	A × L *F* = 1.98 *p* = 0.067
Enucleated	0.91 ± 0.05	0.88 ± 0.06		0.48 ± 0.05	0.21 ± 0.03	0.42 ± 0.04	0.49 ± 0.07	0.33 ± 0.09	G × L *F* = 0.07 *p* = 0.79
									A × G × L *F* = 2.60 *p* = 0.017

These acute experiments show that enucleation at P6 has a profound effect on the activity of the visual cortex. However, the retina is not the only region capable of driving V1 activity at this age, and plasticity to restore normal activity patterns begins rapidly.

### P6 enucleation has modest effect on firing rate and continuity development

**Figure 2.**
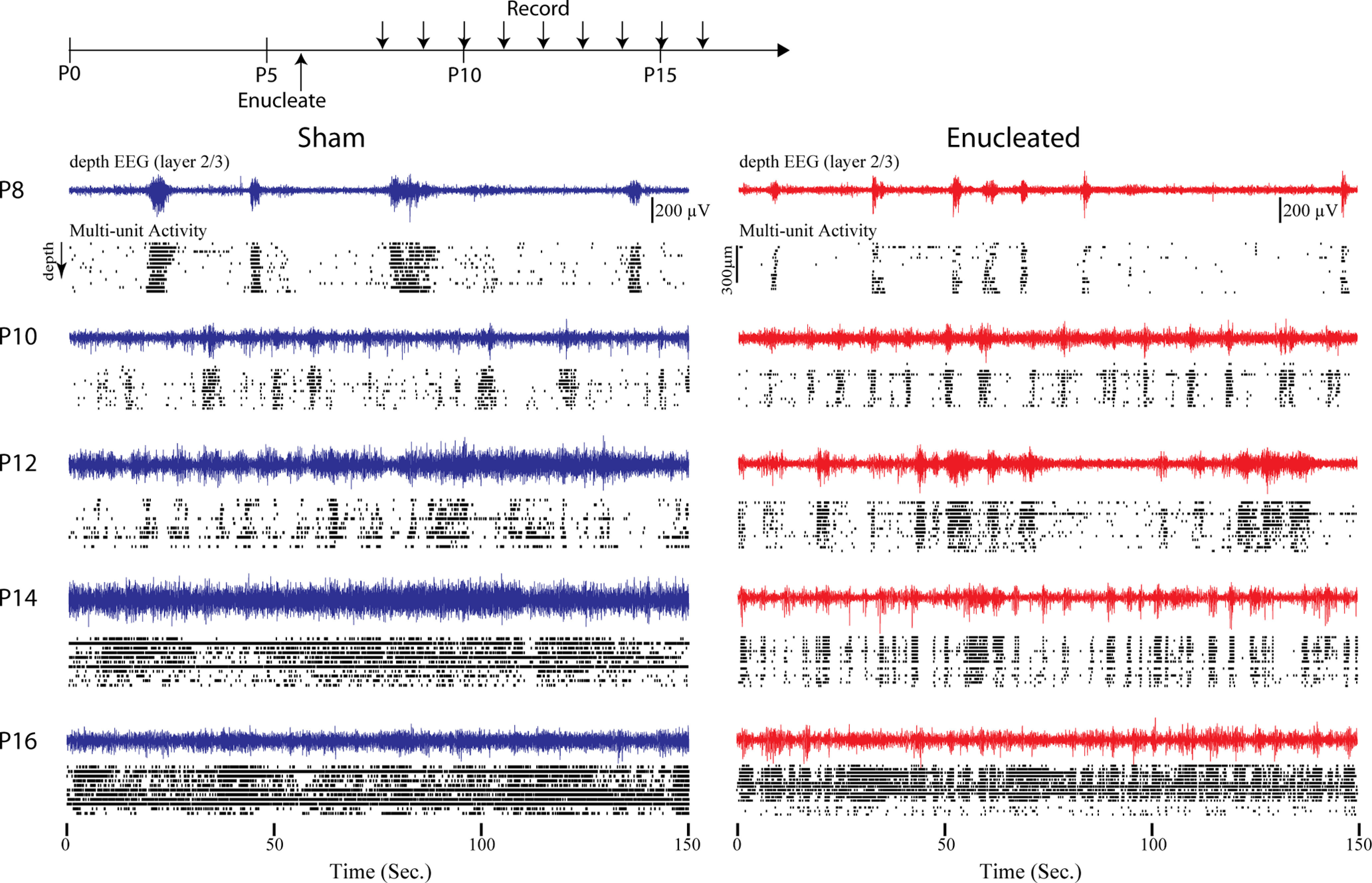
Developmental trajectory of cortical activity following bilateral enucleation. Top, Timing of enucleation and recordings. Recordings are acute and from different animals each day. Below, depth EEG and cleaned multiunit rasters throughout the depth of visual cortex in 2-d intervals. Enucleated animals have some visibly different patterns of activity and silence but total activity develops on a similar trajectory, and enucleated animals resemble sham controls by P16.

To determine the degree to which enucleation changes the developmental trajectory of spontaneous activity, we performed bilateral enucleation or sham surgery on littermates of P6 mice. Spontaneous activity was then acutely recorded from enucleated and sham control littermates at single day intervals between P8 and P17 (Fig. 2). P10–P15 is a time of rapid change in the activity of visual cortex (Shen and Colonnese, 2016). Thus, for quantification we analyzed the following age groups: P8–P9 (*n* = 5 sham and 5 enucleated), P10 (*n* = 4 and 4), P11 (*n* = 2 and 2), P12 (*n* = 4 and 5), P13 (*n* = 4 and 4), P14 (*n* = 5 and 9), P15 (*n* = 5 and 5), and P16–P17 (*n* = 4 and 3).

Qualitatively, spontaneous activity developed similarly in enucleated and sham control animals (Fig. 2). Between P8 and P11, the visual cortex of both groups displayed long periods of silence interrupted by multisecond bursts of rhythmic (8–20 Hz) activity. The bursts of rhythmic activity appeared shorter and occurred at more regular intervals in enucleated animals, particularly between P8 and P11. After P12 the continuity of the spontaneous activity increased in both groups. By P16, significant (>1 s) silent periods were absent in both groups. The detailed trajectory of this development varied between sham and enucleated, however. Between P12 and P15, the activity of enucleated animals consisted of many short bursts of activity, while sham animals showed longer periods of continuous activity, particularly around P14–P15, which was the only age at which the two groups could be reliably distinguished by eye.

**Figure 3.**
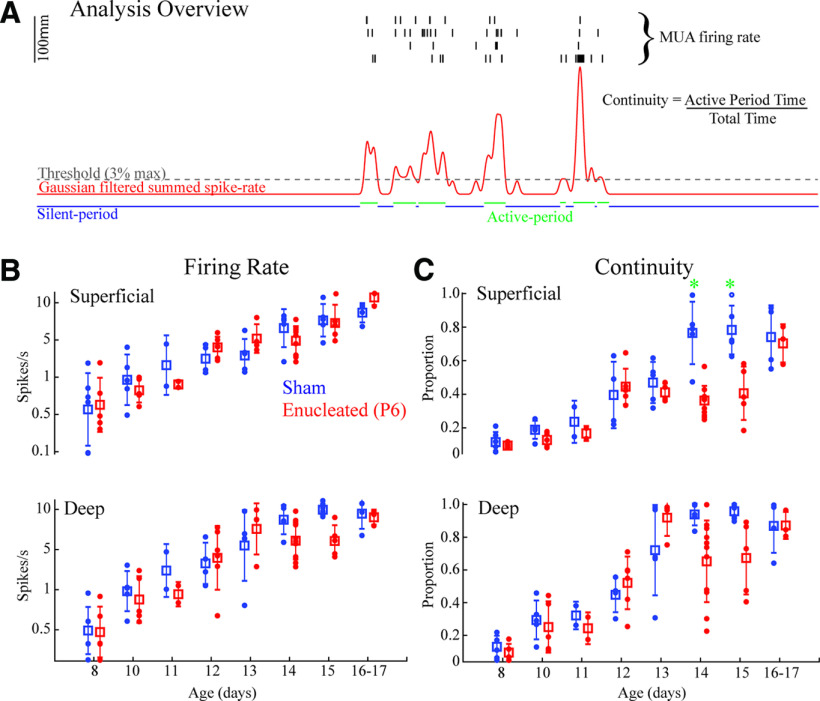
Development of activity in visual cortex is largely independent of retina. ***A***, Schematic of analysis for Figures 3-5, 7. Four channels with maximal multiunit firing are each selected for superficial (L2–L4) and deep (L5–L6) layers and spike times summed. Recordings are divided into silent and active periods based on a threshold of smoothed firing-rate vector for each layer. A general measure of continuity reflects the total amount of time spent in the active “state.” ***B***, Developmental trajectory of multiunit firing rates by age. Mean and SD are shown by box and whiskers, with individual animals as dots. Results for multifactor ANOVA are reported in Table 1. Differences between ages are not shown, but reported in the text. ***C***, Developmental trajectory of continuity. Green asterisks show differences between groups for that age (Tukey’s *post hoc* test *p* < 0.05). Enucleated mice show an initial increase in continuity up to P12, but a second jump at P14 is delayed by 2 d in superficial layers.

We quantified multiunit firing rates in superficial (L2–L4) and deep (L5–L6) layers by age and group in a three-factor ANOVA (Fig. 3*B*; Table 1). This analysis identified a significant effect of age on firing rates, but no significant effect of layer or group, and no interaction among the factors. The effect of age was caused by an increase in firing rates that was roughly log-linear from P8 to P17. *Post hoc* analysis showed that the first day that mean firing rates were significantly different from P8 was P12 for both groups. No significant differences between P12 and older ages were observed for either group. Thus, the developmental trajectory of firing rates is independent of retinal presence, although during normal development retina drives around 80–90% of cortical activity during the first two postnatal weeks (Murata and Colonnese, 2016).

To assay the development of temporal patterning, particularly the continuity of activity, we used multiple measures based on the separation of silent and active periods (Fig. 3*A*). A Gaussian filter (20 ms half-width) was applied to the summed superficial or deep layer MUA and a threshold (3% of peak smooth firing-rate) applied to define silent and active periods.

Continuity was measured as the proportion of total time spent in an active period. Unlike mean firing rates, the developmental trajectory of continuity revealed a complex interaction between retina-dependent and independent influences (Fig. 3*C*). Three-factor ANOVA revealed significant effects for age, group and layer, and significant interactions between age versus group and age versus layer (Table 1). In sham littermates, continuity linearly increased from P8 until reaching an asymptote on P14 in both superficial and deep layers. For both layers, *post hoc* tests first become different from P8 on P13, and further identify significant differences between all ages P14 and older versus P12 and younger. Enucleated animals showed a slightly different trajectory, with both layers increasing their continuity significantly from P8 at P12, 1 d earlier than sham (although absolute continuity was not significantly different between groups at these ages). The biggest difference between groups occurred on P14 when the continuity of superficial layers of enucleated animals did increase as did sham. As a result, superficial layer continuity was significantly different between sham and enucleated animals on both P14 and P15. By P16–P17 the two groups were not significantly different, as continuity in the enucleated superficial layers finally became significantly different from P8–P11 animals (though not a significant jump from P12–P15). Continuity development in the deep layers of both sham and enucleated animals occurred by regular growth from P8 to P13, with no significant differences between groups for any age group. Deep layer continuity in enucleated mice dropped on P14 and P15 after reaching a peak on P13, though this was not significant relative to P13.

Overall, our results suggest that in the superficial layers of mouse visual cortex, continuity develops as a two-step process. First, continuity increases through P13 driven by retinal-independent circuit changes. A second, retina-dependent, increase occurs around P14, though compensatory mechanisms, possibly including continued development of the first process, can complete the development of continuity by P16. Deep layer continuity develops only as a result of the first process, though it may experience subtle alterations in the developmental trajectory following enucleation.

### Retina influences continuity through silent period development

**Figure 4.**
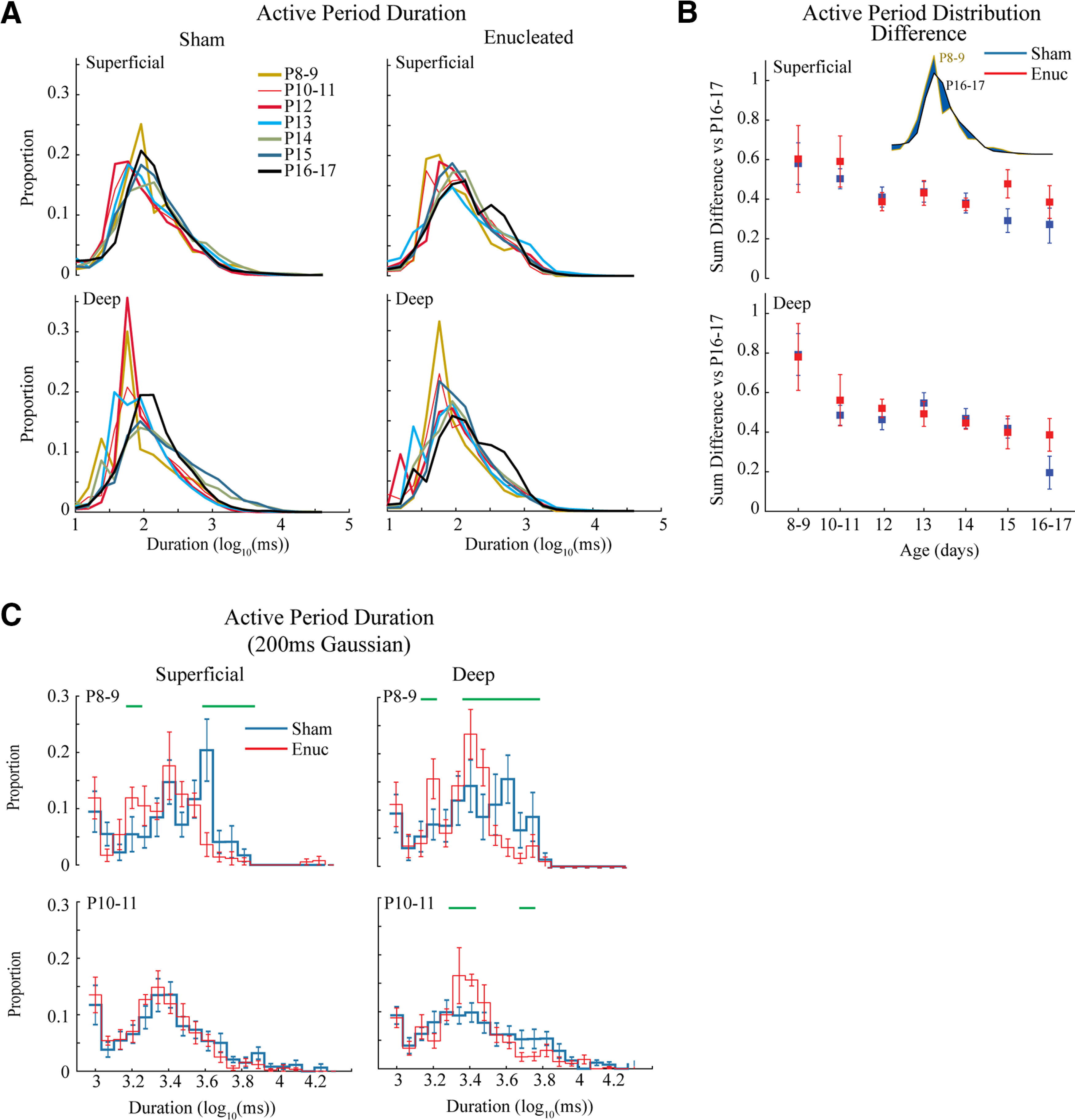
Development of active periods. ***A***, Mean distribution of active period duration by age group. Active period duration is largely similar between ages and treatment groups. ***B***, Quantification of active period duration development. Absolute distance between distributions (shown in inset as blue area) is calculated for all animals in the target age compared with the oldest (P16–P17) group. The mean and 95% confidence intervals (C.I.) for these distances is plotted by age. Results show a steady increase in similarity to P16–P17 for both treatment groups. While an overall effect of group is identified by mANOVA (Table 1), no individual age groups were significantly different between treatments. ***C***, Population mean of active period duration distributions using a longer smoothing filter to eliminate short silent periods. Green lines indicated durations with a significant difference in proportion as determined by permutation analysis (*p* < 0.05).

While the gross measures of firing-rate and continuity showed that development of background activity in the visual cortex is largely independent of retina, visual inspection of the animals suggested that the micropatterning of activity and silences is at least partially retinal dependent (Fig. 2). To capture this difference, we examined the duration of active and silent periods. We first measured the duration of active periods, as defined by the summed MUA activity across superficial or deep layers using a 20-ms Gaussian filter for smoothing (Fig. 3*A*). For this and subsequent analysis we merged the P10 and P11 groups as there was little evidence of difference between them. We observed a steady shift in the active period distributions toward longer periods with age, that was particularly apparent in the deep layers (Fig. 4*A*). To analyze this statistically, the absolute difference between distributions was measured, with P16–P17 animals serving as the reference (Fig. 4*B*). Three-factor ANOVA of these distribution differences showed a significant effect of age and layer and an interaction between the two. We observed an overall effect of enucleation, but no interaction with layer or age; No individual age groups were significantly different between sham and enucleated. This modest effect of active period duration was hard to reconcile with visual inspection of the data, particularly the youngest animals, as sham pups clearly showed long duration events that were not present in the enucleated group (Fig. 2). Because the slow activity transients, driven by retinal waves at these ages, consist of multiple clusters of shorter events, we hypothesized that a wider filter would allow for the capture of these longer events. Therefore, we calculated the activity period duration distributions using a 200-ms half-width Gaussian filter and set a minimum silent period to be 500 ms. At P8–P9 and P10–P11 (this analysis does not work in older, continuous animals), this analysis revealed a reduced occurrence of long duration (>3 s) events in superficial and deep layers at P8–P9 and in deep layers at P10–P11 (Fig. 4*C*). Thus, while the temporal structure of activity “bursts” is largely unchanged following enucleation, long-duration activity periods driven by spontaneous retinal waves are absent when the eyes are removed.

**Figure 5.**
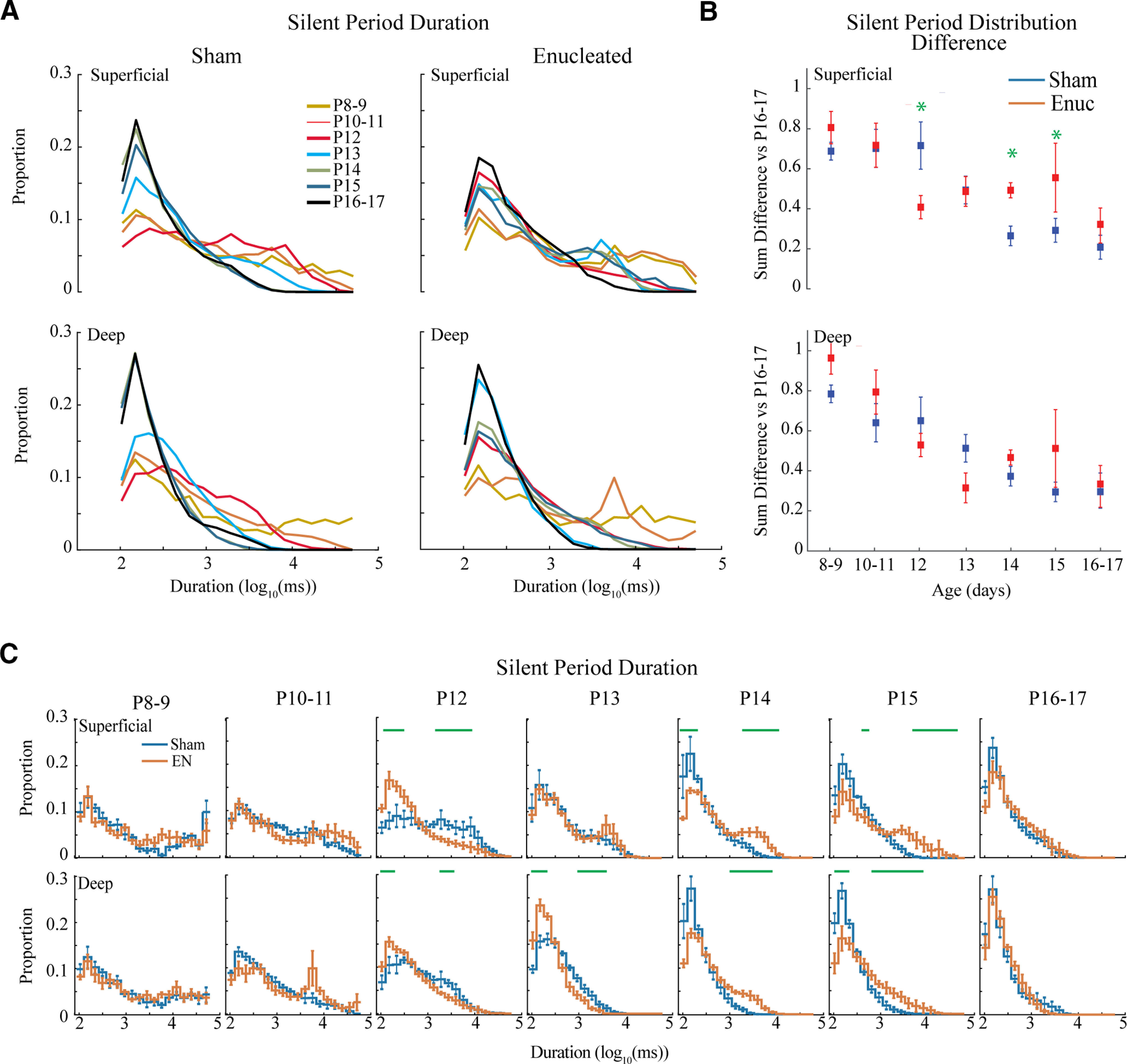
Development of silent periods. ***A***, Population means of the distributions for silent period duration. Both groups and layers show a robust shift from long duration silent periods to short silent periods over the second and third postnatal weeks. ***B***, Mean ± SD of absolute distribution distance from P16–P17 for each age. Green asterisks indicate significant differences between groups at a given age. ***C***, Population means ± SD of silent period duration distribution by age group. Green lines denote durations with significant differences (permutation analysis) between the groups. Enucleated animals show an increase in short duration silent periods 1 d early (P12 vs P13) but then fail to further eliminate long-duration periods at P14 as sham controls. Both groups have eliminated long duration periods by P16–P17.

While active period duration was only modestly modulated by age, silent period duration became clearly shorter during development as long silences were largely eliminated by P16–P17 in both layers (Fig. 5*A*). Quantitative analysis of the distance between silent period distributions for each age (vs P16–P17) showed significant effects of age and group (though not layer), and an interaction between age versus group and between all three factors (Fig. 5*B*; Table 1). *Post hoc* tests revealed silent period durations were different between groups at P12, P14 and P15 in superficial, but not deep layers (Fig. 5*B*). To further explore the origins of these shifts, we directly compared the distributions of sham and enucleated silent periods within each age group (Fig. 5*C*). As predicted by the distance measurements, the distributions of P8–P9 and P10–P11 animals did not show any significant differences. On P12, the silent periods in enucleated animals become shorter than sham in both superficial and deep layers, as shown a by significant increase in the incidence of short periods and a reduction of long periods. On P13, the sham animals shorten their silent periods becoming more like enucleated animals, particularly in the superficial layers. On P14, however, sham animals further shorten their silent periods in both layers, while enucleated animals delay this shortening until P16–P17, when enucleated animals again show no significant differences from sham in either layer.

Together, our active and silent period duration results show the developmental increase in continuity is largely determined by the decreasing length of silent periods. This shift is largely independent of the retina, and likely mediated by the thalamocortical circuit. Interestingly, like total continuity, early absence of the retina results in a shift in the specific timing of certain events, specifically an acceleration of the initial shortening of silent periods and a subsequent delay in a second shortening. However, normal distributions of silent and active periods are achieved within a couple of days, suggesting that retinal-independent mechanisms are sufficient.

### P6 enucleations disrupt early neural oscillation development

**Figure 6.**
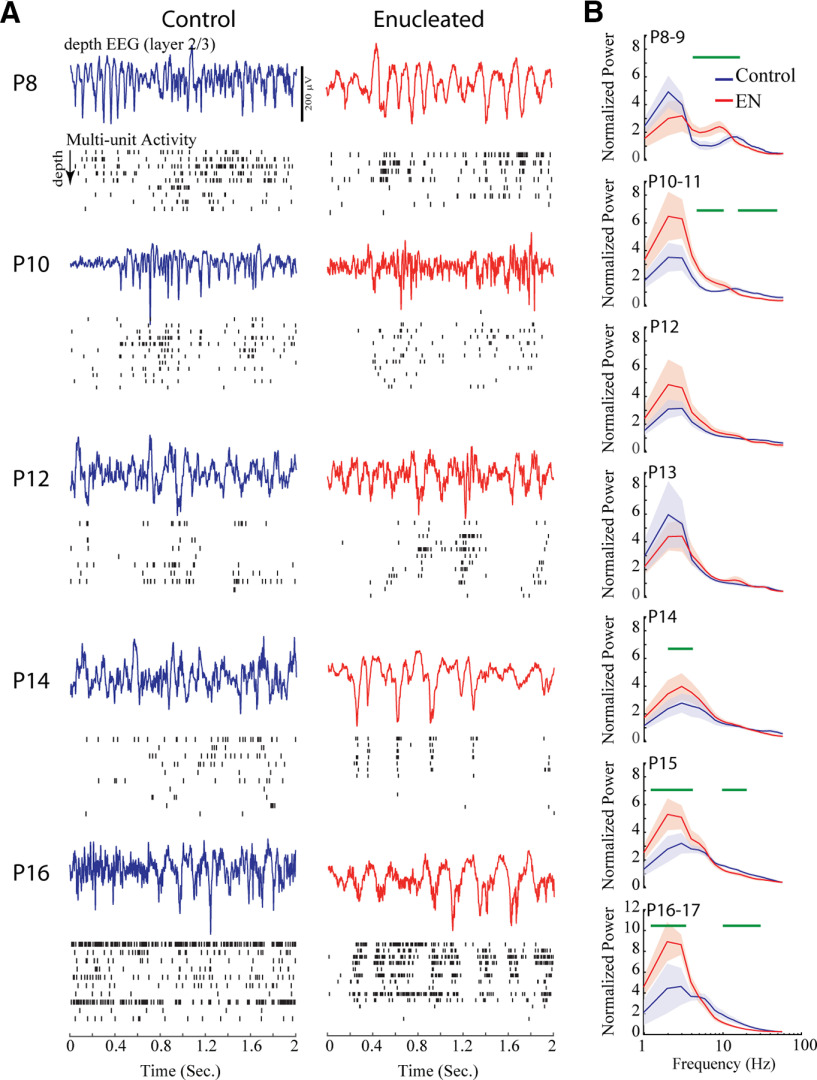
Role of retina in the development of micro-temporal patterning. ***A***, Representative dEEG during active periods and multiunit spike rasters through the depth of cortex. Activity periods were chosen to best represent spectral differences. ***B***, Mean ± SD spectral power distributions by age group. Green lines show frequencies with significant (*p* < 0.05) differences between groups calculated by permutation analysis. Enucleated animals show reduced spindle-burst frequency at P8 and increased power in slow frequencies and reduced high-frequency power beginning at P14.

The normal development of background activity includes a transformation of the micro patterning of activity, as observed in the frequency power of the dEEG. During the first 11 d postnatal, activity in superficial layers of the visual cortex is dominated by spindle-burst oscillations, whose characteristic frequency increases with age (Shen and Colonnese, 2016). These spindle-bursts develop into the broad-band β-γ activity observed during visual activation and wakefulness. Slow-wave activity during behavioral quiescence is first observed at P10 and becomes prominent by P12. To see whether this developmental trajectory is dependent on the retina, we examined the mean power of the layer 2–3 dEEG in sham and enucleated animals (Fig. 6). At P8, active periods included prominent spindle-burst oscillations in both groups. However, the 3- to 10-Hz spectral power was higher in the enucleated group reflecting spindle-bursts that contained oscillations with longer periods compared with control. Mean spectral power in the spindle-burst range peaked near 10 Hz for enucleated animals rather than the 20 Hz seen in sham animals. Normal animals increase the central frequency of spindle-burst oscillations from ∼10 Hz at P6 to 20 Hz at P8 (Shen and Colonnese, 2016), suggesting that the oscillator was frozen at the time of enucleation. At P10–P11, enucleated animals had significantly elevated 4- to 10-Hz power, and reduced 20- to 60-Hz power, suggesting a continued delay in the development of the central generator of spindle bursts. With the switch to the more mature patterns of activity at P12, significant differences in normalized power disappeared. However, beginning on P14 enucleated animals had significantly elevated power at low frequencies and reduced power at high frequencies, reflecting a reduction in the inactivated (slow-wave) state, relative to the activated state.

This analysis of dEEG frequency power confirms our earlier results that the fundamental developmental trajectory of background activity in visual cortex, specifically the switch from spindle-burst oscillations to counterbalanced low and high-frequency oscillations, occurs normally in enucleated animals. Enucleation, however, does result in a moderate delay of the spindle-burst oscillator and some long-lasting changes in the prevalence of slow-waves.

### Effects of P6 enucleation are independent of vision loss

**Figure 7.**
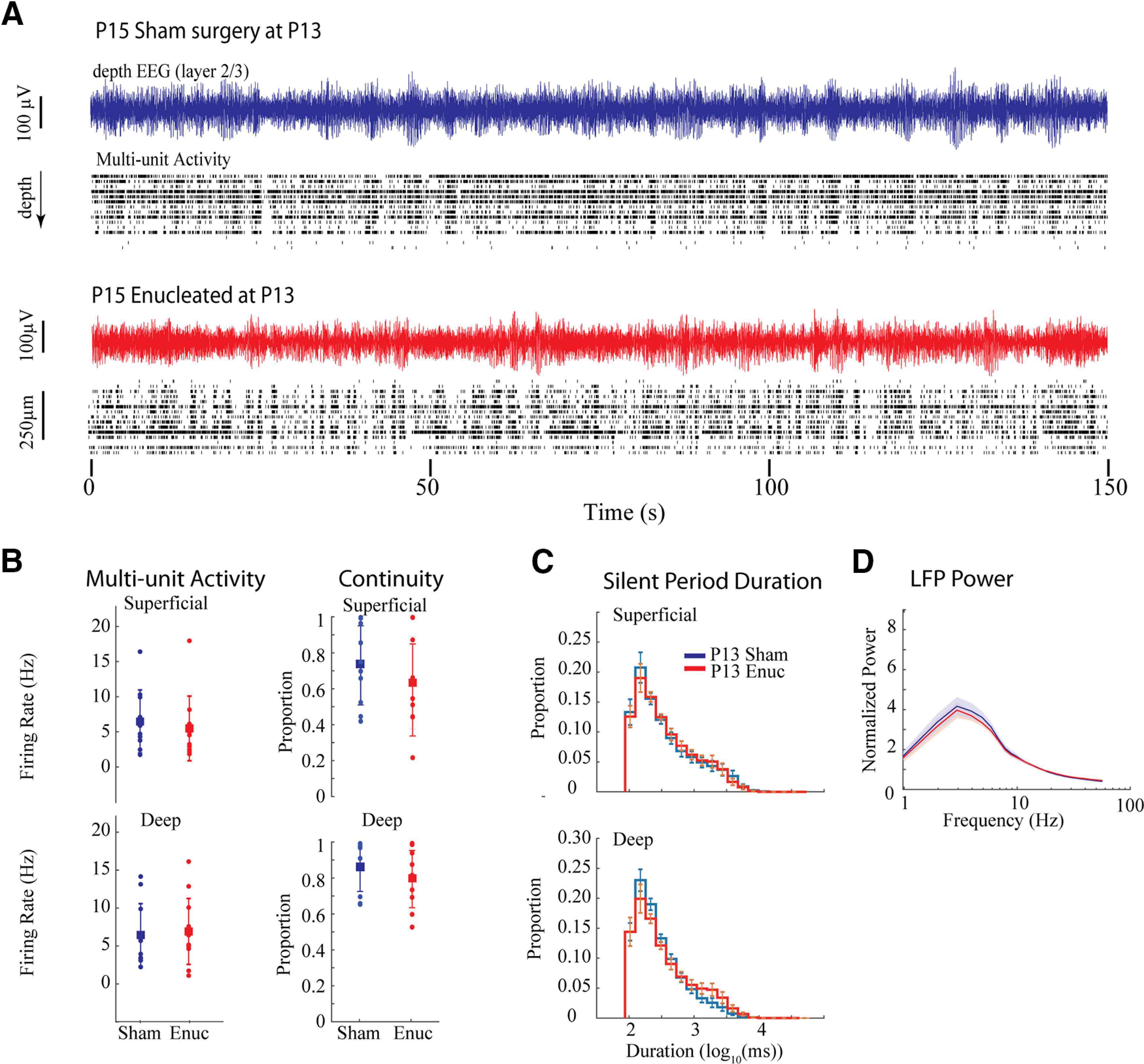
P13 Enucleation does not alter activity at P15. ***A***, Representative example of spontaneous activity for sham control and enucleated animals. ***B***, Population means ± SD for firing-rates and continuity. ***C***, Population means ± SD for silent period duration. ***D***, Population means ± SD for normalized frequency power. No measures show differences between the groups despite differences observed at the same age for P6 enucleated animals.

Our P6 enucleations revealed a two-stage development of continuity, with an initial step occurring on P12 and a second on P14 as well as continuing changes to the slow-wave expression beginning at the same time. Eye-opening in our mice naturally began on P13, suggesting that pattern vision drives the second step. Furthermore, it is possible that the establishment of normal background activity has a critical period which begins only after the end of the early, discontinuous, activity period. To test these hypotheses, we performed enucleations and sham enucleations on littermates at P13 (three litters, *n* = 10 sham, 10 Enuc), after the first switch but before eye-opening. Sham control animals also had their eyelids opened during surgery to coordinate this event. Animals were recorded 2 d later at P15 when continuity, spectral and silent duration differences were most pronounced following P6 enucleation. In contrast to the early enucleations, P13 enucleation had no discernible effect on background activity at P15 (Fig. 7). Multiunit firing rates, continuity, silent period duration and normalized frequency power all showed no significant differences between the sham and enucleated groups. These data show that the onset of visual experience per se is not the driver for the second step in activity development. They further confirm that the acute absence of retina at the time of recording does not cause the differences in activity at P14–P15. The effects of P6 enucleation on activity at P14–P15 are likely the result of compensatory changes in the thalamocortical circuit required to maintain the firing-rate set points.

## Discussion

In this study, we used bilateral enucleation to examine the role that retina and retinal activity plays in the development of spontaneous (“background”) activity of V1. Surprisingly, cortical activity recovers within days of bilateral enucleation at P6 and subsequently develops nearly normally. This occurs although spontaneous activity in the retina is responsible for 80–90% of firing in the visual cortex and thalamus during the first and second postnatal weeks (Fig. 1; Colonnese and Khazipov, 2010; Murata and Colonnese, 2016). Most importantly, the timing of a major developmental switch in thalamocortical activity (Colonnese and Phillips, 2018) is largely unchanged. This switch includes the acquisition of “continuous” background firing, the elimination of development-specific oscillations, and the onset of adult-like frequency distributions indicative of the inhibition-balanced cortical circuit. In this the mammalian cortex appears similar to the zebrafish optic tectum, the network activity patterns of which remain largely intact following binocular enucleation (Avitan et al., 2017; Pietri et al., 2017). Our data confirm that immature spindle-bursts in visual cortex are an intrinsically generated thalamocortical network oscillation (Luhmann and Khazipov, 2018). Under normal circumstances, the duration, occurrence and microstructure of these oscillations arise through an interaction between activity (usually spontaneous) originating in the driving input to thalamus (e.g., retina for dLGN; paralimbic cortex for ventral medial thalamus; Hartung et al., 2016) and the specialized early properties of the thalamocortical circuit (Bitzenhofer et al., 2017). In the absence of the normal driving input, the circuit quickly compensates to generate activity similar to the non-deprived cortex, though at a reduced level (Khazipov et al., 2004). We extend these results by showing that the developmental process that eliminates these early network properties and replaces them with mature sensory processing dynamics, a process that is exquisitely timed to the onset of active sensation (whisking in somatosensory cortex; eye-opening in visual cortex), is largely independent of the sense organ, at least in the visual system. We identified a number of subtle developments–particularly a delay in the onset of fully continuous background activity and the balance between low-frequency and fast (β-γ) activity occurring with eye-opening–that are disrupted by P6 enucleation, but not by P13 enucleation. Thus, changes in retinal activity do not underlie the development of background activity in visual cortex, but the early presence of retina is required to set up circuit conditions required for the exact developmental expression of this activity.

### Thalamocortical homeostatic control of background activity development

Neuronal firing-rates (as well as higher-order structure such as firing-rate correlations and proximity to criticality) are under homeostatic control in more mature juvenile visual cortex, returning to baseline levels within days following eye-lid suture (Hengen et al., 2016; Wu et al., 2020). Before our study it was unclear when and how these homeostatic set points are established. Are spike-rate set-points established by incoming retinal activity levels during early development, or are they an independent function of cortical (or thalamocortical) neurons? Development poses a particularly thorny problem for homeostatic mechanisms as activity is constantly changing. Is this change a result of a changing set-point or is there no set point, with activity determined solely by the inputs? Here, we asked whether the developmental increase in firing-rates (Colonnese et al., 2017) and continuity (Shen and Colonnese, 2016) is timed by retinal inputs or by changing homeostatic set-points within thalamocortex. Our results suggest the latter. This is remarkable because in the intact system, the macro patterning, as well as upwards of 90% of the synaptic drive to thalamus and cortex, is provided by the retina until late in the second postnatal week, when the influence of the retina on cortical activity is reduced (Colonnese et al., 2010; Murata and Colonnese, 2016; Gribizis et al., 2019). However, retinal input is not the sole determinant of cortical firing rates or patterns. Retinal waves actually provide a net de-synchronizing influence to relay thalamus and cortex (Weliky and Katz, 1999; Siegel et al., 2012), with synchronized bursts following rapidly on removal or silencing of the eyes. The thalamic response to retinal waves is amplified fivefold by an excitatory feedback loop with visual cortex (Murata and Colonnese, 2016). Additional amplification and modulation is likely provided by transient circuits formed between subplate, thalamus and overlying cortex (Kanold and Luhmann, 2010) and by inhibitory networks within cortex (Kirmse et al., 2015; Murata and Colonnese, 2020). Thus, there is significant potential by thalamic and local cortical circuits to modulate firing rates within homeostatically set ranges, possibly by the highly synchronous activities remaining after eye removal (Wosniack et al., 2021).

“True” homeostasis requires the capacity for down-regulation following over activation (Turrigiano, 2017), which we did not examine here because of the difficulty in consistently over-activating the developing retina. Thus, it remains unclear whether the consistent shifts in firing-rate and continuity we observed result from a shifting set-point or reflect a changing age-dependent firing-rate maxima that is constantly trying to approach the adult set-point.

In the future, it will be important to understand the extent to which, in normal animals, the increase in continuity and firing-rates are driven by shifts in the underlying firing of the retina, which may in fact change quite significantly and contribute to the normal changes observed during development. In fact, matched developmental shifts in firing-rate and pattern are likely, with the ultimate output of the system determined by a compromise between retinal, thalamic and cortical homeostatic set points. Evidence for this comes from the subtle changes in silent period duration, continuity, and frequency power we observed in the enucleated animals.

### Role of the retina in regulating thalamocortical development

While the developmental trajectory of visual cortex was very similar in P6 enucleated and sham animals, there were some subtle differences in the timing and quality of activity: (1) spindle-burst oscillations did not accelerate between P6 and P10; (2) a second decease in silent-period duration that finalizes the transition to continuous activity was delayed by 2–3 d; (3) background dEEG contained excess low frequency activity that persisted until the end of the study (P16–P17). These changes are likely not a result of the loss of the specific temporal patterning provided by retina as (1) retinal waves do not pattern the rapid oscillations within thalamocortex (Colonnese and Khazipov, 2010); (2) P13 enucleation did not have any effect on these characteristics. Instead, our results suggest that between P6 and P12 retinal absence either prevents circuit maturation or causes circuit adjustments that are required for normal activity after P13. One likely locus for this circuit change is relay thalamus, as the onset of continuous activity originates there and it contributes to the development of dEEG spectral power (Murata and Colonnese, 2019). Eyeless mice experience a precocious and more dense development of cortical feedback connections, which may contribute to premature shortening of silent periods and to the later developmental delay (Seabrook et al., 2013). The reorganization of subplate and interneuron connectivity which occurs in the second postnatal week is another potential locus for aberrant circuit development following P6 enucleation (Lim et al., 2018; Kanold et al., 2019). One process unlikely to contribute is sprouting of other sensory inputs into visual thalamus or thalamic regions to visual cortex, as P6 is after the period when calcium waves establish cortical sensory identity (Moreno-Juan et al., 2017).

### Clinical relevance

Continuous EEG monitoring has been formally recommended for use in all neonates at risk of brain injury by the American Clinical Neurophysiology Society****(Shellhaas et al., 2011). The EEG’s utility is based on the well described, age-linked, changes in the continuity of background activity as well as the stereotyped evolution of spontaneous activity patterns, which are also observed in animal models including mice (Cirelli and Tononi, 2015). The relay thalamus appears to be the central organizer of this development, at least for primary sensory cortex (Murata and Colonnese, 2019). However, whether thalamic activity changes simply reflect changing input or result from maturing thalamic circuits has been unclear. Our data clearly support the latter hypothesis, as removal of the eyes did not significantly alter the timing or form of activity development. Our data would predict that pre thalamic lesions to the primary “driver” input would be difficult to detect by clinical EEG, and further suggest that thalamic lesions should be suspected when there is significant developmental delay or discontinuity in the cortical EEG of infants.

## Synthesis

Reviewing Editor: Arianna Maffei, SUNY Stony Brook Decisions are customarily a result of the Reviewing Editor and the peer reviewers coming together and discussing their recommendations until a consensus is reached. When revisions are invited, a fact-based synthesis statement explaining their decision and outlining what is needed to prepare a revision will be listed below. Note: If this manuscript was transferred from JNeurosci and a decision was made to accept the manuscript without peer review, a brief statement to this effect will instead be what is listed below.

This is a transferred manuscript. The authors provided a satisfactory response to the comments of the reviewers and revised the manuscript accordingly. The study presents novel results highlighting the role of homeostatic mechanisms to stabilize cortical activity during postnatal development that act independently of the driving input. These results presented here, while descriptive, are impactful and open important questions for future studies.

## References

[B1] Ackman JB, Crair MC (2014) Role of emergent neural activity in visual map development. Curr Opin Neurobiol 24:166–175. 10.1016/j.conb.2013.11.011 24492092 PMC3957181

[B2] André M, Lamblin MD, d’Allest AM, Curzi-Dascalova L, Moussalli-Salefranque F, S Nguyen The T, Vecchierini-Blineau MF, Wallois F, Walls-Esquivel E, Plouin P (2010) Electroencephalography in premature and full-term infants. Developmental features and glossary. Neurophysiol Clin 40:59–124. 10.1016/j.neucli.2010.02.002 20510792

[B3] Avitan L, Pujic Z, Molter J, Van De Poll M, Sun B, Teng H, Amor R, Scott EK, Goodhill GJ (2017) Spontaneous activity in the zebrafish tectum reorganizes over development and is influenced by visual experience. Curr Biol 27:2407–2419.e4. 10.1016/j.cub.2017.06.056 28781054

[B4] Bitzenhofer SH, Ahlbeck J, Wolff A, Wiegert JS, Gee CE, Oertner TG, Hanganu-Opatz IL (2017) Layer-specific optogenetic activation of pyramidal neurons causes beta-gamma entrainment of neonatal networks. Nat Commun 8:14563. 10.1038/ncomms14563 28216627 PMC5321724

[B5] Cirelli C, Tononi G (2015) Cortical development, electroencephalogram rhythms, and the sleep/wake cycle. Biol Psychiatry 77:1071–1078. 10.1016/j.biopsych.2014.12.017 25680672 PMC4444390

[B6] Cohen MX (2014) Analyzing neural time series data: theory and practice. Cambridge: MIT Press.

[B7] Colonnese MT (2014) Rapid developmental emergence of stable depolarization during wakefulness by inhibitory balancing of cortical network excitability. J Neurosci 34:5477–5485. 10.1523/JNEUROSCI.3659-13.2014 24741038 PMC3988407

[B8] Colonnese MT, Khazipov R (2010) Slow activity transients in infant rat visual cortex: a spreading synchronous oscillation patterned by retinal waves. J Neurosci 30:4325–4337. 10.1523/JNEUROSCI.4995-09.2010 20335468 PMC3467103

[B9] Colonnese MT, Phillips MA (2018) Thalamocortical function in developing sensory circuits. Curr Opin Neurobiol 52:72–79. 10.1016/j.conb.2018.04.019 29715588 PMC6139060

[B10] Colonnese MT, Kaminska A, Minlebaev M, Milh M, Bloem B, Lescure S, Moriette G, Chiron C, Ben-Ari Y, Khazipov R (2010) A conserved switch in sensory processing prepares developing neocortex for vision. Neuron 67:480–498. 10.1016/j.neuron.2010.07.015 20696384 PMC2946625

[B11] Colonnese MT, Shen J, Murata Y (2017) Uncorrelated neural firing in mouse visual cortex during spontaneous retinal waves. Front Cell Neurosci 11:289. 10.3389/fncel.2017.00289 28979189 PMC5611364

[B12] Demas J, Eglen SJ, Wong RO (2003) Developmental loss of synchronous spontaneous activity in the mouse retina is independent of visual experience. J Neurosci 23:2851–2860. 12684472 10.1523/JNEUROSCI.23-07-02851.2003 PMC6742078

[B13] Dereymaeker A, Pillay K, Vervisch J, De Vos M, Van Huffel S, Jansen K, Naulaers G (2017) Review of sleep-EEG in preterm and term neonates. Early Hum Dev 113:87–103. 10.1016/j.earlhumdev.2017.07.003 28711233 PMC6342258

[B14] Froudarakis E, Fahey PG, Reimer J, Smirnakis SM, Tehovnik EJ, Tolias AS (2019) The visual cortex in context. Annu Rev Vis Sci 5:317–339. 10.1146/annurev-vision-091517-034407 31525143 PMC7485906

[B15] Gramsbergen A (1976) The development of the EEG in the rat. Dev Psychobiol 9:501–515. 10.1002/dev.420090604 1001836

[B16] Gribizis A, Ge X, Daigle TL, Ackman JB, Zeng H, Lee D, Crair MC (2019) Visual cortex gains independence from peripheral drive before eye opening. Neuron 104:711–723.e3. 10.1016/j.neuron.2019.08.015 31561919 PMC6872942

[B17] Hanganu-Opatz IL, Butt SJB, Hippenmeyer S, De Marco Garcia NV, Cardin JA, Voytek B, Muotri AR (2021) The logic of developing neocortical circuits in health and disease. J Neurosci 41:813–822. 33431633 10.1523/JNEUROSCI.1655-20.2020 PMC7880298

[B18] Harris KD, Thiele A (2011) Cortical state and attention. Nat Rev Neurosci 12:509–523. 10.1038/nrn3084 21829219 PMC3324821

[B19] Hartung H, Brockmann MD, Pöschel B, De Feo V, Hanganu-Opatz IL (2016) Thalamic and entorhinal network activity differently modulates the functional development of prefrontal-hippocampal interactions. J Neurosci 36:3676–3690. 10.1523/JNEUROSCI.3232-15.2016 27030754 PMC6601737

[B20] Hengen KB, Torrado Pacheco A, McGregor JN, Van Hooser SD, Turrigiano GG (2016) Neuronal firing rate homeostasis is inhibited by sleep and promoted by wake. Cell 165:180–191. 10.1016/j.cell.2016.01.046 26997481 PMC4809041

[B21] Iyer KK, Roberts JA, Metsäranta M, Finnigan S, Breakspear M, Vanhatalo S (2014) Novel features of early burst suppression predict outcome after birth asphyxia. Ann Clin Transl Neurol 1:209–214. 10.1002/acn3.32 25356399 PMC4184550

[B22] Kanold PO, Luhmann HJ (2010) The subplate and early cortical circuits. Annu Rev Neurosci 33:23–48. 10.1146/annurev-neuro-060909-153244 20201645

[B23] Kanold PO, Deng R, Meng X (2019) The integrative function of silent synapses on subplate neurons in cortical development and dysfunction. Front Neuroanat 13:41. 10.3389/fnana.2019.00041 31040772 PMC6476909

[B24] Khazipov R, Sirota A, Leinekugel X, Holmes GL, Ben-Ari Y, Buzsáki G (2004) Early motor activity drives spindle bursts in the developing somatosensory cortex. Nature 432:758–761. 10.1038/nature03132 15592414

[B25] Kirmse K, Kummer M, Kovalchuk Y, Witte OW, Garaschuk O, Holthoff K (2015) GABA depolarizes immature neurons and inhibits network activity in the neonatal neocortex in vivo. Nat Commun 6:7750. 10.1038/ncomms8750 26177896

[B26] Kummer M, Kirmse K, Zhang C, Haueisen J, Witte OW, Holthoff K (2016) Column-like ca(2+) clusters in the mouse neonatal neocortex revealed by three-dimensional two-photon ca(2+) imaging in vivo. Neuroimage 138:64–75. 10.1016/j.neuroimage.2016.05.050 27222218

[B27] Leighton AH, Lohmann C (2016) The wiring of developing sensory circuits-from patterned spontaneous activity to synaptic plasticity mechanisms. Front Neural Circuits 10:71. 10.3389/fncir.2016.00071 27656131 PMC5011135

[B28] Lim L, Mi D, Llorca A, Marín O (2018) Development and functional diversification of cortical interneurons. Neuron 100:294–313. 10.1016/j.neuron.2018.10.009 30359598 PMC6290988

[B29] Luhmann HJ, Khazipov R (2018) Neuronal activity patterns in the developing barrel cortex. Neuroscience 368:256–267. 10.1016/j.neuroscience.2017.05.025 28528963

[B30] McCormick DA, Nestvogel DB, He BJ (2020) Neuromodulation of brain state and behavior. Annu Rev Neurosci 43:391–415. 10.1146/annurev-neuro-100219-105424 32250724 PMC12237593

[B31] Mitra P, Bokil H (2007) Observed brain dynamics. New York: Oxford University Press, USA.

[B32] Moreno-Juan V, Filipchuk A, Antón-Bolaños N, Mezzera C, Gezelius H, Andres B, Rodríguez-Malmierca L, Susín R, Schaad O, Iwasato T, Schüle R, Rutlin M, Nelson S, Ducret S, Valdeolmillos M, Rijli FM, López-Bendito G (2017) Prenatal thalamic waves regulate cortical area size prior to sensory processing. Nat Commun 8:14172. 10.1038/ncomms14172 28155854 PMC5296753

[B33] Murata Y, Colonnese MT (2016) An excitatory cortical feedback loop gates retinal wave transmission in rodent thalamus. Elife 5:e18816. 10.7554/eLife.18816 27725086 PMC5059135

[B34] Murata Y, Colonnese MT (2018) Thalamus controls development and expression of arousal states in visual cortex. J Neurosci 38:8772–8786. 10.1523/JNEUROSCI.1519-18.2018 30150360 PMC6181317

[B35] Murata Y, Colonnese MT (2019) Thalamic inhibitory circuits and network activity development. Brain Res 1706:13–23. 10.1016/j.brainres.2018.10.024 30366019 PMC6363901

[B36] Murata Y, Colonnese MT (2020) GABAergic interneurons excite neonatal hippocampus in vivo. Sci Adv 6:eaba1430. 10.1126/sciadv.aba1430 32582852 PMC7292633

[B37] Olavarria JF, Hiroi R (2003) Retinal influences specify cortico-cortical maps by postnatal day six in rats and mice. J Comp Neurol 459:156–172. 10.1002/cne.10615 12640667

[B38] Pachitariu M, Steinmetz N, Kadir S, Carandini M, Harris KD (2016) Kilosort: realtime spike-sorting for extracellular electrophysiology with hundreds of channels. bioRxiv. doi: 10.1101/061481.

[B39] Pavlidis E, Lloyd RO, Boylan GB (2017) EEG - a valuable biomarker of brain injury in preterm infants. Dev Neurosci 39:23–35. 10.1159/000456659 28402972

[B40] Pietri T, Romano SA, Pérez-Schuster V, Boulanger-Weill J, Candat V, Sumbre G (2017) The emergence of the spatial structure of tectal spontaneous activity is independent of visual inputs. Cell Rep 19:939–948. 10.1016/j.celrep.2017.04.015 28467907 PMC5437726

[B41] Raichle ME (2010) Two views of brain function. Trends Cogn Sci 14:180–190. 10.1016/j.tics.2010.01.008 20206576

[B42] Renart A, de la Rocha J, Bartho P, Hollender L, Parga N, Reyes A, Harris KD (2010) The asynchronous state in cortical circuits. Science 327:587–590. 10.1126/science.1179850 20110507 PMC2861483

[B43] Rossant C, Kadir SN, Goodman DFM, Schulman J, Hunter MLD, Saleem AB, Grosmark A, Belluscio M, Denfield GH, Ecker AS, Tolias AS, Solomon S, Buzsaki G, Carandini M, Harris KD (2016) Spike sorting for large, dense electrode arrays. Nat Neurosci 19:634–641. 10.1038/nn.4268 26974951 PMC4817237

[B44] Seabrook TA, El-Danaf RN, Krahe TE, Fox MA, Guido W (2013) Retinal input regulates the timing of corticogeniculate innervation. J Neurosci 33:10085–10097. 10.1523/JNEUROSCI.5271-12.2013 23761904 PMC3682386

[B45] Seabrook TA, Burbridge TJ, Crair MC, Huberman AD (2017) Architecture, function, and assembly of the mouse visual system. Annu Rev Neurosci 40:499–538. 10.1146/annurev-neuro-071714-033842 28772103

[B46] Shellhaas RA, Chang T, Tsuchida T, Scher MS, Riviello JJ, Abend NS, Nguyen S, Wusthoff CJ, Clancy RR (2011) The american clinical neurophysiology society’s guideline on continuous electroencephalography monitoring in neonates. J Clin Neurophysiol 28:611–617. 10.1097/WNP.0b013e31823e96d7 22146359

[B47] Shen J, Colonnese MT (2016) Development of activity in the mouse visual cortex. J Neurosci 36:12259–12275. 10.1523/JNEUROSCI.1903-16.2016 27903733 PMC5148222

[B48] Siegel F, Heimel JA, Peters J, Lohmann C (2012) Peripheral and central inputs shape network dynamics in the developing visual cortex in vivo. Curr Biol 22:253–258. 10.1016/j.cub.2011.12.026 22264606

[B49] Stevenson NJ, Oberdorfer L, Koolen N, O’Toole JM, Werther T, Klebermass-Schrehof K, Vanhatalo S (2017) Functional maturation in preterm infants measured by serial recording of cortical activity. Sci Rep 7:12969–12963. 10.1038/s41598-017-13537-3 29021546 PMC5636845

[B50] Tiriac A, Blumberg MS (2016) The case of the disappearing spindle burst. Neural Plast 2016:8037321. 10.1155/2016/8037321 27119028 PMC4826930

[B51] Tononi G, Cirelli C (2020) Sleep and synaptic down-selection. Eur J Neurosci 51:413–421. 10.1111/ejn.14335 30614089 PMC6612535

[B52] Tuge H, Kanayama Y, Chang HY (1960) Comparative studies on the development of EEG. Jpn J Physiol 10:211–220. 10.2170/jjphysiol.10.211 13839778

[B53] Turrigiano GG (2017) The dialectic of hebb and homeostasis. Philos Trans R Soc Lond B Biol Sci 372:20160258. 28093556 10.1098/rstb.2016.0258 PMC5247594

[B54] Uddin LQ (2020) Bring the noise: reconceptualizing spontaneous neural activity. Trends Cogn Sci 24:734–746. 10.1016/j.tics.2020.06.003 32600967 PMC7429348

[B55] van Rooij LG, Toet MC, Osredkar D, van Huffelen AC, Groenendaal F, de Vries LS (2005) Recovery of amplitude integrated electroencephalographic background patterns within 24 hours of perinatal asphyxia. Arch Dis Child Fetal Neonatal Ed 90:245. 10.1136/adc.2004.064964 PMC1721875 15846017

[B56] Vanhatalo S, Kaila K (2006) Development of neonatal EEG activity: from phenomenology to physiology. Semin Fetal Neonatal Med 11:471–478. 10.1016/j.siny.2006.07.008 17018268

[B57] Wallois F, Routier L, Heberle C, Mahmoudzadeh M, Bourel-Ponchel E, Moghimi S (2020) Back to basics: the neuronal substrates and mechanisms that underlie the electroencephalogram in premature neonates. Neurophysiol Clin 51:5–33. 33162287 10.1016/j.neucli.2020.10.006

[B58] Weliky M, Katz LC (1999) Correlational structure of spontaneous neuronal activity in the developing lateral geniculate nucleus in vivo. Science 285:599–604. 10.1126/science.285.5427.599 10417392

[B59] Whitehead K, Pressler R, Fabrizi L (2017) Characteristics and clinical significance of delta brushes in the EEG of premature infants. Clin Neurophysiol Pract 2:12–18. 10.1016/j.cnp.2016.11.002 30214965 PMC6123866

[B60] Wosniack ME, Kirchner JH, Chao LY, Zabouri N, Lohmann C, Gjorgjieva J (2021) Adaptation of spontaneous activity in the developing visual cortex. Elife 10:e61619. 10.7554/eLife.61619 33722342 PMC7963484

[B61] Wu YK, Hengen KB, Turrigiano GG, Gjorgjieva J (2020) Homeostatic mechanisms regulate distinct aspects of cortical circuit dynamics. Proc Natl Acad Sci USA 117:24514–24525. 10.1073/pnas.1918368117 32917810 PMC7533694

